# Blockchain in Healthcare: Insights on COVID-19

**DOI:** 10.3390/ijerph17197167

**Published:** 2020-09-30

**Authors:** Antonio Fusco, Grazia Dicuonzo, Vittorio Dell’Atti, Marco Tatullo

**Affiliations:** 1 Department of Economics, Management and Business Law, University of Bari Aldo Moro, 70124 Bari, Italy; info@studioantoniofusco.it (A.F.); grazia.dicuonzo@uniba.it (G.D.); vittorio.dellatti@uniba.it (V.D.); 2 Department of Basic Medical Sciences, Neurosciences and Sense Organs, University of Bari ALDO MORO, 70124 Bari, Italy

**Keywords:** COVID-19, healthcare management, global health, blockchain, artificial intelligence

## Abstract

The SARS-CoV2 pandemic has impacted risk management globally. Blockchain has been increasingly applied to healthcare management, as a strategic tool to strengthen operative protocols and to create the proper basis for an efficient and effective evidence-based decisional process. We aim to validate blockchain in healthcare, and to suggest a trace-route for a COVID19-safe clinical practice. The use of blockchain in combination with artificial intelligence systems allows the creation of a generalizable predictive system that could contribute to the containment of pandemic risk on national territory. A SWOT analysis of the adoption of a blockchain-based prediction model in healthcare and SARS-CoV-2 infection has been carried out to underline opportunities and limits to its adoption. Blockchain could play a strategic role in future digital healthcare: specifically, it may work to improve COVID19-safe clinical practice. The main concepts, and particularly those related to clinical workflow, obtainable from different blockchain-based models have been reported here and critically discussed.

## 1. Background

The World Health Organization (WHO) has recommended that countries worldwide draw up a “Pandemic Plan”, due to the increased possibility of pandemic risk. A Pandemic Plan is typically developed according to the pandemic phases declared by the WHO and aims to achieve clear results in managing pandemics from the early stages [1].

In healthcare, we may recognize different approaches in preparing for an emergency; in fact, each emergency is characterized by different phases: mitigation, preparation, response, and recovery [2]. The “tabletop exercise” is a useful tool that simulates the emergence of a critical situation; it provides a scenario that benefits from both communication and cooperation between different sectors and areas, such as management, workers, logistics, communication, and finance. A proper approach could provide a general framework and a mental model reproducing the perfect environment for future decision making [2]. 

On 30 January 2020, the World Health Organization (WHO) announced a public health emergency due to the spreading of a new coronavirus called SARS-CoV2, associated with the COVID-19 disease, and on 11 March 2020 the epidemic became a pandemic [3]. 

The COVID-19 outbreak is demonstrating the vulnerability of worldwide people towards novel and highly contagious biological agents. In this landscape, several countries have considered the reinforcement of strategies of “risk management” as a priority. 

The main concern of timely risk management is related to data sharing among clinicians and mass media, as in most cases this may create panic in the general population. However, in some countries, healthcare authorities are used to block or delay the sharing of important data to preventive understanding of the risk to which people are exposed and also to properly limit the diffusion of dangerous pathologies. In recent decades, the growth of diagnostic technologies and biomedical devices have aided the industrialized countries in strengthening and standardizing risk management in healthcare [4]. 

The SARS-CoV2 pandemic has involved all continents, testing risk management in all the main worldwide institutions [5]: in this context, Wendelboe et al. have conceived and designed a specific tabletop exercise for universities and companies to suggest reliable objectives and detailed instructions in order to prevent and manage COVID-19 infection:

(i) Analysis of cases of COVID-19 with known travel-related exposure,
(ii) Analysis of cases of COVID-19 with no known (i.e., community) exposure,
(iii) Outbreak of COVID-19 in the local region,
(iv) Recovery from COVID-19 (phases 2 and 3) and “looking ahead” [6].

A standardized plan to guide and modulate the communication between institutions and medical staff is strategic for disseminating the information that the community needs, because people should be adequately prepared for the emergency, and trained to improve their skills and preparation [7].

In Italy, the SARS-CoV2 pandemic started in February 2020, from “patient zero” living in Codogno, a small town in the north of Italy. The Lombardy region, involved from the start in the management of the epidemic, has proved to be able to make a rapid response to the outbreak in the north of Italy; in less than two weeks, a traditional infectious disease department was converted into a “COVID-19 department”, doubling bed capacity and creating a sub-intensive ward and a highly-targeted care ward. This healthcare plan was further improved, thanks to the efforts of many clinicians, nurses, administrative staff, and hospital management, over a very short time. In fact, in just five days, the COVID-19 department was expanded, separating the first floor from the remaining floors to allow operators to move freely. In just ten days, the ground floors of the COVID-hospitals were converted into an emergency are, where patients with specific symptoms were evaluated and treated with a proper and safe protocol [8].

In February 2020, the Netherlands also became involved in the COVID-19 outbreak. The Dutch national epidemic management (DNEM) team met in March to discuss limitations and understand the spread in the entire country. The strategy was to prevent and manage a hypothetical community infection: sampling of different health-workers from the main hospitals was used to allocate additional professionals in specific areas of the country (North Brabant and Limburg). The Netherlands carried out a rapid two-day study of nine hospitals to observe the health of professionals working in these areas of the country, alerting local authorities when they showed mild respiratory symptoms [9]. Hospitals were asked to provide the screening test to operators and this process represented a representative sample to investigate. Thanks to these data, the regional authorities decided to use restrictive measure to limit the infection to a large part of the population [10].

From these two experiences, it is clear how the risk management of pandemics has preserved European countries from a more severe diffusion of the infection. Adequate containment measures, proportionate to the evolution of the epidemiological situation, based on the pandemic plans drawn up according to WHO directives, have prevented unpredictable health risk and have coordinated national response to the medical emergency [11].

Healthcare management can use several strategic tools to be effective: data sharing and data mining, machine learning, artificial intelligence, and blockchain are the most impacting strategies [12]. In recent years, blockchain technology has been increasingly applied to healthcare, to strengthen the operative protocols and to create the proper basis for an efficient and effective evidence-based decisional process. Blockchain plays a strategic role in safely sharing data between groups of persons, independently of the reliability and the cross-checking of these groups. Blockchain usually works by collaborative tools and can be used in a new workflow or in improved protocols with particular attention to risk management. We aim to validate blockchain in healthcare, and, in more detail, to suggest a traceroute for a COVID19-safe clinical practice.

## 2. Blockchain in Healthcare Management

Blockchain technology belongs to the wider category of Distributed Ledger technologies, whose functioning is based mainly on a register structured in blocks linked in a network; each transaction performed in a block of the network is validated through a process based on the consensus distributed across all the nodes (that is the devices/users connected to the net).

The transactions represent the result of the operations that occur among the subjects within the network. Each block, through a cryptographic system, maintains a reference to the previous one, hence the concept of blockchain. Blockchain is not filed on a centralized server, as happens in traditional web applications, but it is distributed on devices (computers) of the network (called nodes), each containing a copy of the whole blockchain. Moreover, it is useful to highlight for our analysis two relevant aspects which characterize this kind of technology: (i) the decentralization of consensus and (ii) decentralization of the ledgers.

Due to the decentralization of consensus, the existence of trustworthiness among the subjects involved in any kind of transaction and a central authority may no longer be necessary [5,7]. Similarly with the second aspect, the repetition and the saving of different copies of different blockchains across the nodes of the network guarantees greater security of the system and equity among the users, who can access the same information simultaneously and, therefore, the traceability and immutability of the validated transactions contained in the blocks. Therefore, blockchain is a peer-to-peer network in which all the participants in the network can trust in the system without necessarily trusting each other.

The reference literature highlights the application of this type of technology in different sectors, such as the financial, credit, insurance, commerce, and agri-food sectors for the reorganization of specific processes [13,14,15].

Blockchain applied to the health sector can offer new and effective opportunities to improve several activities associated with the prevention and control of pathologies and, therefore, better clinical risk management in the context of a pandemic emergency such as the current one. The sudden appearance and the rapid and uncontrolled diffusion around the world of Coronavirus has shown us not only the failure of existent healthcare surveillance systems in promptly managing the public health emergency, but also an evident lack of advanced predictive systems based on the sharing of clinical data on a large scale, able to prevent or at least lessen emergencies of such magnitude.

Different studies suggest the application of blockchain in the health sector mainly for sharing and better management of patients’ data, electronic health records (EHR) and, if less frequently, the supply chain management of medical devices and drugs, the management of drug prescriptions, to improve the scientific research and the divulging of scientific knowledge, and for the development of precision medicine [16,17,18,19,20].

The development of new and smart approaches to medicine has opened new pathways for innovative procedures, which have been demonstrated to work well and safely [21,22].

The use of technology can allow the exchange of healthcare data: this is an important step towards the effective interoperability among different Electronic Health Records (EHR) system. The management of EHR with blockchain technology may reduce clinical bias, thus improving the overall healthcare outcomes [23]. The issue of interoperability among different EHR systems may be overcome by using separate blockchain systems that would work as a bridge to ensure cross-communication: in more detail, we may operate with two main blockchain users that will communicate through a third, in the middle of the two cross-talking blockchains. Blockchain is an opportunity to ensure cryptographically secured data exchanges between two or more users: recently, this opportunity has created interest within the scientific community, which aims mainly to facilitate interaction between different secured networks; this will ensure a trustworthy decentralization of activities like asset and message exchange [21,22,23].

The acquisition, conservation, and sharing of clinical data would also foster the development of precision medicine and, therefore, the personalization of prevention, diagnosis, and treatment for the single patient (patient-focused care).

Smart contracts based on blockchain technology can also be used to automatize auditing processes, improve the supply chain management of pharmaceutical products and verify their quality and compliance with current regulations [23]. Moreover, current IT infrastructures do not facilitate the constant sharing of the results of scientific research and clinical studies and this does not foster the development and sharing of scientific research capital. Blockchain can be a valid instrument of knowledge management that promotes the diffusion of the best clinical practices and evidence-based medicine [24].

However, the decentralized and transparent nature of this technology raises, in some contexts of application, issues linked to privacy protection of the patient and network security (with many aspects still unsolved and subject to debate), with special focus on the sharing of sensitive data in public blockchains.

The management of health records through the use of this technology is based on the possibility of sharing the data among the different parties involved in healthcare management, preserving at the same time patients’ privacy, security, and the immutability of data and information contained in the blockchain workflow. In this specific case healthcare providers and healthcare institutions aim at:

(i) building a predictive model (machine learning) through the analysis of electronic health records or clinical data related to particular or rare pathological cases;
(ii) using the data, conveniently re-elaborated, to predict healthcare outcomes.

This kind of instrument contributes, therefore, to the wider process of clinical risk management, allowing healthcare organizations to prevent and contain the onset of adverse events [20,25].

**Figure 1:**
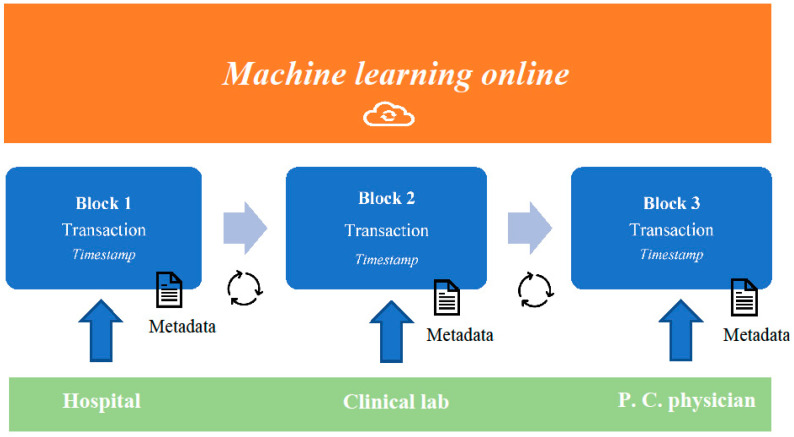
The concept of a blockchain-based predictive model.

It is believed that the combination of blockchain and machine learning systems will be able to generate data, usable to create predictive models that are useful in risk management: blockchain is based on technologies that offer the strategic advantage of a distributed (peer-to-peer), immutable and safe ledger, and privacy protections for the patients. Recently, researchers have developed medical applications involving the use of the internet: such applications were based on an artificial intelligence that was able to promote continuous machine learning to improve critical steps of diagnosis and treatment of several diseases [24]. The data transmitted by the users/healthcare providers in the blockchain are not sensitive data of the patients, but anonymized data and anonymized information that remains usable on each server for healthcare providers. Furthermore, users may participate in research networks to extrapolate big data, aimed at creating, for example, predictive models of medical workflow or pandemic onset and development. Such models may be processed by machine learning systems, updated through an interactive process of information exchanges within the network. This model would be updated and tested until it achieves the highest reliability. At this point, the machine-learning system stops, and the last updated model is identified as the consensus model [25,26] (Figure 1).

The use of blockchain and its combination with artificial intelligence systems allows the creation of a generalizable predictive system that, included in the wider risk management process, could contribute decisively to the containment of pandemic risk on national territory.

The results of a constantly updated predictive model, based on information on and clinical data of patients, can in particular influence not only clinical practice but more generally the programmatic policies of risk containment at regional and national levels.

### 2.1. SWOT Analysis of the Adoption of the Blockchain-Based Prediction Model in Healthcare

**Figure 2:**
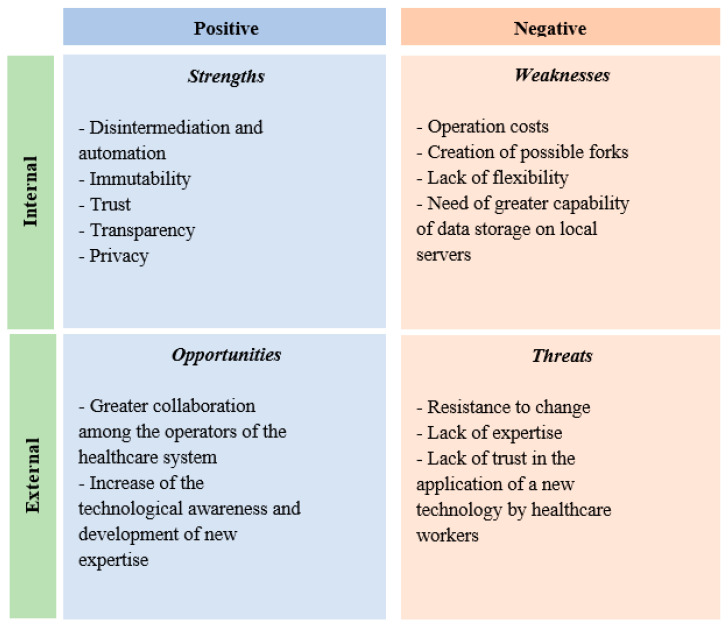
SWOT analysis of the adoption of the blockchain-based model in healthcare.

To better understand, examine and identify the main strengths and weaknesses of the represented model, the offered opportunities and threats, a SWOT analysis that underlines the opportunities and limits of adoption has been realized (Figure 2).

The disintermediation, intended as the absence of a central authority that collects, processes and validates the data or the built and shared models, allows the reduction of time, errors and costs in the performance of processes, aiming at the construction and update of a predictive model which supports clinical practice and risk management. The blockchain is an integrated system and the processes implied in it are automatized and standardized [27,28,29].

The transactions validated through the blockchain and the data contained are immutable, in the sense that they cannot be modified or eliminated, and this guarantees their authenticity, increasing at the same time the safety of the environment in which the operations occur [27,29,30,31].

Moreover, the cryptographic system, the immutability of the data distributed in the whole network and the absence of a centralized authority generates greater trust in the system, as the need to keep this among the parties involved in the process disappears [31,32]. The commitment among the parties in the chain to collaborate in the processing and update of the partial models is justified by the common interest in obtaining an increasingly accurate, functional, and effective predictive model [29,30].

All participants can verify the operations that occur in the network as they have a copy of the whole blockchain on their device and this makes the process transparent [30]. The sharing of whole copies of the blockchain, in a model in which sensitive data on a single patients are shared, would create many problems linked to the compliance with privacy regulations, especially if organizations other than public healthcare companies participate in the network [33,34,35]. For example, each participant (healthcare provider, institution, etc.) would have the problem of identifying the subject responsible for any illegal activity committed in violation of privacy regulations. Considering, for example, privacy regulations disciplined by the General Data Protection Regulation (GDPR) that represents a regulation law on data protection and privacy in the European Union (EU), a very important aspect is represented by the modality of detecting the owners or those responsible for the data processing and of guaranteeing, at the same time, protections provided by the same regulations for the parties involved in the network [27,28,29].

However, there are many other aspects linked to the theme of privacy to consider for the regular users of this kind of technology [29,32]. Thus, if decentralization and immutability, typical characteristics of the blockchain, allow on the one hand the actual transparency and safety of transactions, on the other hand this can create a point of conflict with the regulations in force [36,37,38].

To face these problems, the examined model does not include the entry of patients’ direct and sensitive data, but specifically metadata (hash, flags, errors of the models) and partial predictive models. Thus, regulatory issues linked to the privacy protection could be solved and become a strong point in the implementation of the examined model.

### 2.2. SWOT Analysis of the Adoption of the Blockchain-Based Prediction Model in SARS-CoV-2 Infection

From the earliest data on the clinical manifestations of SARS-CoV-2 infection provided by Chinese scholars, information was not homogeneous and was misleading in the very first stage of the contagion [38,39,40]. Initially, affected people were reported to have an average age of 49–56 years, with the rare involvement of the pediatric population [39,40,41]. In a second stage, the spread of COVID-19 was controlled and monitored using swabs for respiratory fluids of nasopharyngeal origin on which the presence of the virus was tested. This specific examination was administered by several hospitals and research centers in Asian countries and it was often contested as not being efficient for proper and rapid detection of the virus.

The SWOT applies to this specific condition, as the improvements to be applied are various and at different parts of the medical process. In some cases, researchers have also used integrated SWOT–AHP (Analytic Hierarchy Process) analysis in other fields, to identify strengths, weaknesses, opportunities and threats (SWOT factors), and to weight the factors identified according to the AHP method. [42,43] On the other hand, SWOT analysis is commonly used to describe case studies, comparing them to the related literature, acting as a kind of decision-maker in order to go beyond a “best approach” [43].

The strengths of blockchain in such conditions are the disintermediation and automation of the information chain, the immutability of the information, and the reliability and transparency of the information obtained in all interested countries with respect for people’s privacy. On the other hand, several opportunities may also be developed within this outbreak: the first of these is the opportunity to reinforce teambuilding and international networking among regions of different countries.

Given the low specificity of the swab test, the opportunity to increase the technological awareness of healthcare personnel may also develop new expertise to better approach the COVID-19 pandemic; for confirmation of the diagnosis of new coronavirus infection, it has been necessary to carry out a laboratory test, the Real-Time PCR (RT-PCR), on respiratory samples and serum. The use of RT-PCR is the most reliable technique, even if there is a slight possibility of false-positive results. In agreement with the positive improvements of SWOT indications, the discovery and development of oligonucleotide primers and probes against the SARS-CoV-2 viral genome has allowed the RT-PCR to be successful, although coronavirus may undergo frequent mutations of its genome.

The SARS-CoV-2 genome sequences were discovered and deposited in public databases to develop safe and universal molecular diagnostics in a short time. Scholars from the Berlin Institute of Virology have developed assays to distinguish SARS-CoV-2 from SARS-CoV infection, based on the nucleotide sequence of the RNA-dependent RNA polymerase gene (RdRp). Cross-reactivity was validated through the use of several known respiratory pathogens from infected clinical samples. Now it is the most used and validated protocol to declare positivity. This protocol has been validated and confirmed by the World Health Organization [44,45]. This was possible thanks to the genetic relationship between the 2003 SARS-CoV infection and modern synthetic nucleic acid technologies.

After these earlier developed and tested protocols, several different protocols based on reverse polymerase chain reaction (RT-PCR) have been used to confirm COVID-19 infection. Gene sequencing is strategic for validating any type of PCR test; Cepheid and Sherlock Biosciences have recently developed an alternative test based on Clustered Regularly Interspaced Short Palindromic Repeats (CRISPR) technology, used not only in genetic editing, but also for its diagnostic potential, and already used for the diagnosis of Zika virus [46].

Furthermore, research into treatments that can combine early healing and less biological and economic cost have pushed researchers towards experimentation with smart materials and nanotechnologies, even if the main issues regarding the safe applications of such technologies on human patients remain [47,48,49].

To provide precise information on the epidemic trend in real-time, an informatic system should be easy to use and quick to achieve results. Fatally, information flow without a standardized workflow, like the blockchain-based protocols, may result in being altered or misunderstood between two different users; nevertheless, blockchain-based protocols are able to ensure privacy and limited sharing of patients’ information. Blockchain is structurally aimed to work in environments where trust in data is better than in physical subjects. In fact, data registered in blockchain workflow are impossible to be changed or altered. In this landscape, it is necessary to observe that in a blockchain of permission type (consortium), the management and authority in defining access, control, authorization and especially the possibility to add transactions to the distributed ledger is only attributed to one specific group of operators (who act as validators) [50]. Thus, only a selected group of nodes can participate in the process of distributed consensus. The use of this typology of governance would contribute not only to the resolution of problems linked to privacy, but would particularly adapt to the objectives of the model.

As is well known, each node (user) of the chain has on his device a copy of the whole blockchain and this aspect, if on the one hand it creates undeniable advantages in terms of certainty and safety of the datum and transactions, on the other hand it generates, especially in the case of big networks, greater costs for the management and storage of data [32]. Moreover, the creation of new blocks could generate latencies of transmission in the network due to the bandwidth at the time of block validation (fork). Thus, the new blocks could reach the nodes at different moments, in fact generating temporary inconsistencies in the blockchain, which constitute a limitation [51].

The immutability of the blockchain represents one of the main advantages in the use of technology: however, this characteristic can represent a limitation when the modification of a transaction is necessary [52].

The use of a predictive model based on innovative technologies such as blockchain and machine learning and the awareness of the benefits that derive from use inevitably generate the development of new applications and competences [32].

However, blockchain technology is still evolving and thus it must face important social challenges, such as cultural change. Accepting and adopting an innovative technology that implies a method of work completely different from the traditional could generate, within different organizational contexts, strong resistance to change. Moreover, because of the low rate of adoption of similar models in the healthcare sector, expertise able to draw up and make operational models of this type in the short term has not developed in Italy. If on the one hand this could obstruct the implementation of a system, in the short term, on the other hand, it could become food for thought in starting a process of adoption of this kind of tool, precisely because of the current situation of emergency.

Finally, it is worth highlighting that the implementation of a model that brings into relation a bigger possible number of healthcare providers and institutions (at national level and/or single regional territory level) can undoubtedly be beneficial to the whole community as it would increase the degree of integration of risk management policies among the operators of the whole healthcare system, except for current regional autonomies in healthcare management.

### 2.3. Translational Applications of Blockchain-Based Outcomes and Workflow in Healthcare

The combination of the potentialities of blockchain and those expressed by artificial intelligence systems, such as machine learning, is undoubtedly an innovative and effective approach for the construction of models able to rapidly identify choices of diagnosis and treatment specific for COVID-19 patients, contributing also to the formation/development of clinical guidelines for possible future epidemics similar to coronavirus.

**Figure 3:**
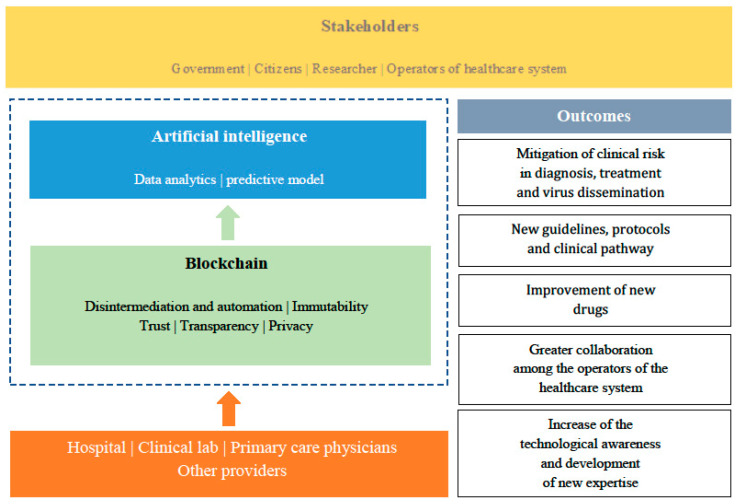
Insights into the main outcomes derived from the blockchain-based model.

All the data originating from healthcare providers (e.g., clinical laboratories, hospitals, primary care physicians and pediatricians) and other sources, can be collected and shared while respecting privacy and security through blockchain and later analyzed using solutions based on artificial intelligence. Such a system is a valid tool for risk management, useful in the phases of diagnosis and treatment of the patient affected by COVID19, but also necessary for research into more suitable therapies concerning the typology of the patient and the risk and health conditions associated (comorbidity, associated risk factors, etc.), as well as to foster the development of new drugs or increasingly adequate diagnostic and therapeutic protocols [53] (Figure 3).

**Figure 4:**
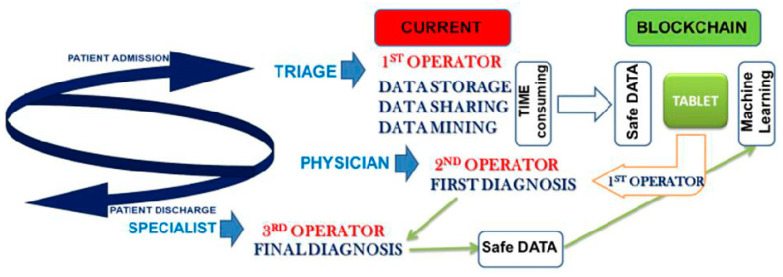
Insights into the clinical workflow derived from the blockchain-based model.

Hence, the patient becomes the protagonist on an alternative path to the current one, in which the protection, the analysis, and the reprocessing of data are not standardized and not predictable in large populations. In the path supported by the management model inspired and based on blockchain, triage is entirely computerized and managed by self-implementing systems of machine-learning, with a verification system through feedback of the diagnosis at admission; this allows a reduction of time-consuming procedures and a rationalization of the storage of sensitive information, which are managed by only by those few authorized to perform data analysis. Data-mining and data-storage are finally transmitted through a certified data flow, free from methodological and interpretative errors (operator’s bias), that are treated according to certification and standard modulations, and are finally processed in a worldwide network which makes the single datum vital for the creation of databases useful for the future management of medical data via artificial intelligence that can support traditional medicine (Figure 4).

## 3. Conclusions

Data sharing and data mining, machine learning, artificial intelligence, and blockchain are the most impacting current strategies for healthcare management. The blockchain is increasingly applied to healthcare to create the proper basis for an efficient and effective evidence-based decisional process. Blockchain is a valid approach to safely sharing data between groups of persons, independently of the reliability and the cross-checking of these groups. Blockchain can be used in a new workflow or in improved protocols with particular attention to risk management. Based on this, we can say that blockchain plays a strategic role in healthcare, in particular for COVID19-safe clinical practice.
